# Exploring the Diagnostic Utility of Tear IgE and Lid Wiper Epitheliopathy in Ocular Allergy Among Individuals with Hay Fever

**DOI:** 10.3390/ijms26189116

**Published:** 2025-09-18

**Authors:** Rinu Thomas, Serap Azizoglu, Cenk Suphioglu, Ereeny Mikhail, Moneisha Gokhale

**Affiliations:** 1 School of Medicine (Optometry), Faculty of Health, Deakin University, Waurn Ponds, Geelong, VIC 3216, Australia; s223602085@deakin.edu.au (R.T.); serap.azizoglu@deakin.edu.au (S.A.); e.mikhail@deakin.edu.au (E.M.); 2 NeuroAllergy Research Laboratory (NARL), School of Life and Environmental Sciences, Faculty of Science, Engineering and Built Environment, Deakin University, Waurn Ponds, Geelong, VIC 3216, Australia; cenk.suphioglu@deakin.edu.au; 3 Institute for Mental and Physical Health and Clinical Translation (IMPACT), Deakin University, Waurn Ponds, Geelong, VIC 3216, Australia; 4 Centre for Sustainable Bioproducts (CSB), Deakin University, Waurn Ponds, Geelong, VIC 3216, Australia

**Keywords:** ocular allergy, hay fever, tear biomarkers, lid wiper epitheliopathy, allergic rhinitis, seasonal allergy

## Abstract

Allergic rhinitis (hay fever) prevalence has increased in Australia. People with hay fever often experience many eye symptoms, especially itching. This study explores clinical correlations between tear IgE levels and ocular allergy signs in hay fever sufferers, focusing also on eyelid wiper friction damage from eye rubbing. In a cross-sectional study from November 2024 to January 2025, 16 individuals with self-reported hay fever and 17 healthy controls were recruited. Participants completed demographic and allergy-related questionnaires, including symptoms and quality of life assessments. Tear samples were analyzed for IgE and MMP-9 biomarkers. Ocular surface parameters-bulbar redness, palpebral roughness, and lid wiper epitheliopathy (LWE)-were graded. Corneal and conjunctival dendritic cells were also evaluated. Elevated tear IgE significantly correlated with self-reported hay fever, QUICK score, MiniRQLQ, eye rubbing frequency, and lower LWE grade. The hay fever group showed significantly higher LWE compared to healthy controls (*p* < 0.001), indicating frictional eyelid damage. ROC analysis of tear IgE yielded an AUC of 0.893 (cut-off 0.03 IU/mL; sensitivity 90%, specificity 85%). Tear IgE is a useful biomarker for ocular inflammation and may indicate friction-related eyelid damage in allergy sufferers. Incorporating LWE grading into clinical assessments of ocular allergy is recommended.

## 1. Introduction

Hay fever, also known as Allergic Rhinitis (AR), is highly prevalent in Australia, affecting 23.8% of adults, with the highest rates reported in Victoria at 29.7% [1]. Ocular Allergy (OA), a common Type I hypersensitivity disorder, often coexists with hay fever and is triggered by allergens such as pollen, dust mites, animal dander, and fungal spores [2]. In Victoria, grass pollen is typically the primary trigger for this allergic event [3]. Over the years, there has been an earlier onset and longer duration of the core pollen season in Melbourne [4], which has impacted the duration and intensity of the hay fever season. OA encompasses a spectrum of conditions, including Seasonal Allergic Conjunctivitis (SAC), Perennial Allergic Conjunctivitis (PAC), Atopic Keratoconjunctivitis (AKC), Vernal Keratoconjunctivitis (VKC), Contact Blepharoconjunctivitis (CBC), and Giant Papillary Conjunctivitis (GPC). SAC is the major focus of this study, as it is one of the milder forms of seasonal allergy and is challenging to detect amongst its comorbidities.

Approximately 40–60% of individuals with allergies report ocular symptoms, including itching, inflammation, tearing, burning, dryness, and the sensation of a foreign body [5,6]. These symptoms are central to the clinical diagnosis of OA according to the American Academy of Ophthalmology (AAO) diagnostic criteria for allergic conjunctivitis (AC) [7]. Ocular itch is the hallmark symptom of OA [8], often prompting eye rubbing and leading to the disruption of the tear film homeostasis [9] and alteration of tear biochemistry [10]. Persistent friction due to eye rubbing exacerbates ocular surface damage, leading to Lid Wiper Epitheliopathy (LWE), corneal nerve injury [11], increased surface roughness [12,13], and a heightened risk of developing keratoconus, which is a progressive thinning of the cornea [14,15,16,17,18,19]. Vigorous eye rubbing induces microscopic damage to ocular surfaces due to mechanical friction between the sliding surfaces (eyelids, cornea, and conjunctiva) [20,21]. Eye rubbing also has a key impact on the alteration of ocular biochemistry, with an increase in the expression of proteins relating to inflammation, immune response, and cellular development [10,22]. Hence, eye rubbing and frictional damage to the ocular surface in OA sufferers must be diagnosed and managed effectively to reduce associated trauma to the ocular surfaces.

While there is growing evidence supporting the usefulness of tear IgE in diagnosing ocular allergies among hay fever sufferers, there are still no robust studies validating tear IgE as a stand-alone diagnostic tool without comparison to serum IgE. The ability of tear IgE to reliably differentiate individuals with hay fever from healthy controls remains incompletely understood. Moreover, OA is a multifactorial disease with several comorbidities. The damage to the lid wiper, which is the first point of contact on the ocular surface during eye rubbing, has not been reported in previous studies that focus on OA. This highlights the potential usefulness of LWE assessment, in combination with tear IgE measurement, in improving the diagnostic evaluation of OA among hay fever sufferers.

Given that SAC and PAC are Immunoglobulin E (IgE)-mediated hypersensitivity reactions, measuring tear IgE offers a more direct and reliable biomarker for OA diagnosis, when compared to the traditional serum IgE assessment [23,24]. In addition to IgE, other inflammatory markers found in tears, such as Matrix Metalloproteinase-9 (MMP-9), known to play a role in corneal wound healing, have proven valuable in detecting ocular surface inflammation associated with tear film dysfunction [25], keratoconus [26], dry eye disease [27], and contact lens wear [28]. The collection of tear samples and, the need for prompt storage and transportation to the laboratory for analysis have historically been challenging. The recent advances in diagnostic technology, such as i-ImmunDx^TM^ tear IgE POCT [23], now enable the rapid, non-invasive, and precise analysis of minuscule tear volumes. These innovations make tear-based immunological testing more practical and accessible in clinical settings, as demonstrated by this research.

This study aims to translate these diagnostic advances into clinical practice by identifying a correlation between subjective and objective optometric measures with tear IgE, thereby improving the diagnosis and management of OA in individuals with hay fever. In addition, this study will evaluate the frictional aspect of OA and its impact on eyelid wipers.

## 2. Results

### 2.1. Participants’ Details

The total number of participants enrolled in the study was 46. Of these, 7 participants were excluded due to contact lens use, which could influence clinical grading and friction-related parameters. An additional 6 participants were excluded, as they reported only perennial allergies due to pets, dust, or mold, with no hay fever. The final sample consisted of 33 participants: 16 in the hay fever group and 17 in the healthy control group. The statistical power calculated post hoc for this sample size was 89% (mean hay fever group = 10.8 ± 14.5, mean HCs group = 0.18 ± 0.14, effect size 1.0) using G* Power 3.1.9.7. The distribution of the allergy status of the participants is illustrated in the pie chart of Figure S1. A total of 16 participants (6 males and 10 females) with an average age of 34.4 ± 8.5 years in the hay fever group and 17 participants (4 males and 13 females) with an average age of 31.2 ± 6.9 years in the control group met the inclusion criteria and completed the study with no significant difference in age distribution between the groups (*p* = 0.16, Mann–Whitney U test). Family history of any type of allergy was similar between the hay fever and control groups, with 6 out of 16 participants in the hay fever group and 5 out of 17 in the control group reporting a positive history (*p* = 0.709, Mann-Whitney U test).

### 2.2. History of Use of Medications

Among the 16 hay fever participants, 2 reported never using medications for their hay fever symptoms, while 14 reported using treatments, such as antihistamines, nasal sprays, inhalers, eye drops, or herbal treatments. Among the 14 participants who used treatments, 75% of the participants purchased their medicines over the counter, and 25% of them purchased the medication on prescription. Additionally, 1 participant with hay fever also reported undergoing immunotherapy for Australian grass pollen at present, and 1 participant had completed the immunotherapy treatment in 2018. The medications used by participants in this study are shown in Table S1.

### 2.3. Characteristics of Eye Rubbing

The eye rubbing frequency score for hay fever participants is shown in Figure S2. It is noted that 87.6% of hay fever participants engage with eye rubbing: 50% occasionally, 25% often, and 12.6% more often rub their eyes. Only 2 among the 16 hay fever participants reported no rubbing of eyes during the hay fever season. Among participants in the hay fever group, 50% were observed to rub the outer corners of their eyelids, involving the lid wiper region. Of these, half engaged in soft rubbing, and 64.3% reported that the duration of rubbing lasted only a few seconds, as shown in Figure S3.

### 2.4. Clinical Measurements

The clinical findings, including symptom score, QoL assessments, tear biomarkers, and clinical signs, are presented in Table 1. The median (IQR) of tear IgE levels measured using the i-ImmunDx^TM^ Analyzer was significantly elevated (*p* < 0.001, Mann–Whitney U test) in the hay fever group (2.03 (16.4)) compared to the HCs (0.15 (0.2)). Also, statistically significant differences were observed in the eye rubbing frequency scores, symptom scores, QoL, presence of prominent dendritic cells, and palpebral roughness grade. Interestingly, the LWE grade of the lower eyelid was significantly higher in the hay fever group compared to the HCs.

### 2.5. Clinical Correlation Between Tear IgE and Clinical Findings

Spearman correlations found a strong significant correlation between self-reported hay fever and tear IgE levels (rho = 0.68, *p* < 0.001), palpebral roughness (rho = 0.60, *p* < 0.001), eye rubbing frequency score (rho = 0.60, *p* < 0.001), MiniRQLQ score (rho = 0.78, *p* < 0.001), QUICK score (rho = 0.74, *p* < 0.001), and lower LWE (rho = 0.65, *p* < 0.001) (Table S2).

Tear IgE levels significantly correlated with:

- Eye rubbing frequency score (rho = 0.63, *p* < 0.001);
- MiniRQLQ score (rho = 0.59, *p* < 0.001);
- QUICK score (rho = 0.55, *p* < 0.001);
- Lower LWE (rho = 0.41, *p* = 0.01).

**Figure 1:**
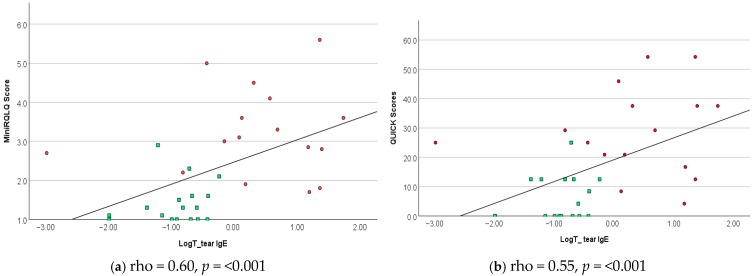
**Correlation between IgE and Questionnaire scores:** The scatter plot illustrates the correlation between tear biomarker, symptom score, and QoL scores. (**a**,**b**) show correlation between subjective symptom scores and IgE levels (*p* < 0.001, n = 33). Red dots represent hay fever group, and green squares represent HC.

No significant correlation was found between tear IgE levels and bulbar redness, palpebral roughness, presence of dendritic cells, upper LWE grade, NIKBUT, tear MMP-9, family history of any allergies, age, or sex. Additionally, strong correlations were observed between the two validated questionnaires: the MiniRQLQ and QUICK scores (rho = 0.84, *p* < 0.001). The scatter plot (Figure 1a,b) shows the correlation between the LogT IgE levels and QUICK and MiniRQLQ scores.

### 2.6. Eye Rubbing and LWE

The eye rubbing frequency score and LWE are, respectively, subjective and objective findings that reflect the presumed frictional impact on the ocular surface. It has been found that the eye rubbing frequency score (*p* = 0.001) and the LWE of the lower lid (*p* < 0.001) were increased significantly among hay fever sufferers compared to HCs. The upper lid LWE also showed an increase in those with hay fever; however, this increase was not statistically significant.

Significant correlations were observed between the following parameters:

- LWE lower lid and:
  ▪ Bulbar redness (rho = 0.60, *p* < 0.001);
  ▪ Presence of dendritic cells (rho = 0.54, *p* = 0.005);
  ▪ MiniRQLQ scores (rho = 0.51, *p* = 0.003);
  ▪ LWE of upper lid (rho = 0.50, *p* = 0.004); and
  ▪ QUICK scores (rho = 0.47, *p* = 0.006).
- Eye rubbing frequency scores and:
  ▪ ▪ MiniRQLQ scores (rho = 0.54, *p* = 0.001); and
  ▪ QUICK scores (rho = 0.60, *p* < 0.001).

### 2.7. ROC

**Figure 2:**
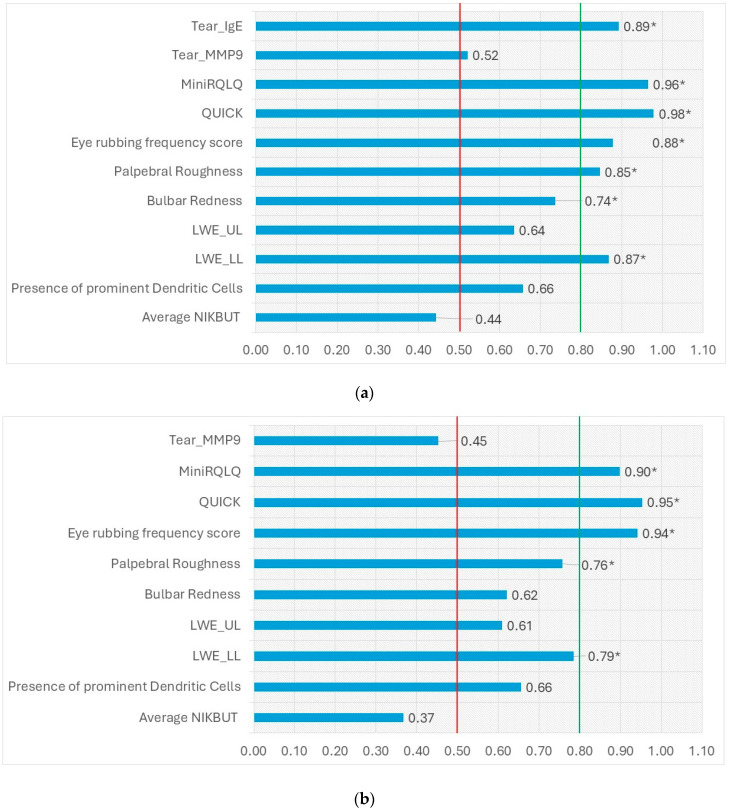
**Overall model quality** (**a**): showing the AUC while grouping participants based on self-reporting of symptoms is the discriminator, (**b**): showing the AUC while grouping participants based on tear IgE values (cut off: 1 IU/mL). * Represents significance levels where *p* < 0.049. AUC values greater than 0.50 (red line) are considered of clinical utility, while values greater than 0.80 (green line) indicate perfect discrimination.

The ROC curve was analyzed to compare the performance of our battery of tests in discriminating between hay fever and healthy controls (Table S3 and Figure 2a). The tear IgE, Mini RQLQ score, QUICK score, palpebral roughness, bulbar redness, lower LWE, and eye rubbing frequency score were significant and acceptable discriminators for those classified based on self-reported symptoms of hay fever and healthy controls. Most importantly, tear IgE showed excellent sensitivity (0.90) and specificity (0.85) to differentiate hay fever sufferers from their healthy controls.

Similarly, the predictive ability of tear IgE to distinguish between subjects with hay fever (tear IgE > 1 IU/mL) and healthy controls (tear IgE < 1 IU/mL) was analyzed (Table S4 and Figure 2b). The eye rubbing frequency score, MiniRQLQ score, QUICK score, palpebral roughness, and lower LWE showed greater AUC and were significant discriminators between groups.

## 3. Discussion

Diagnosis of seasonal allergic conjunctivitis (SAC) is challenging due to its milder form with no corneal involvement and its coexistence with other pathologies, such as dry eye, contact lens discomfort, and other severe forms of OA [29]. This condition presents with severe ocular itch, bulbar redness, and palpebral roughness, which are the major hallmarks in diagnosing OA. However, diagnosing OA may be confused clinically with dry eye, infections, and ocular surface disorders due to the heterogeneous nature of signs and symptoms [29].

This study aimed to develop and evaluate the correlation of tear IgE with subjective and objective clinical OA measures in hay fever sufferers. In addition, this is the first study to report the frictional impact of eye rubbing on the eyelids by assessing LWE in hay fever sufferers. By integrating both subjective and objective assessments, including tear biomarkers in conjunction with validated OA symptoms, and QoL questionnaires, this study sought to translate diagnostic advances into practical tools for routine eye care.

### 3.1. Clinical Predictors of OA

This study uncovered compelling clinical features that strongly and significantly distinguished individuals with hay fever and healthy controls, offering exciting possibilities for streamlined practical diagnostic indicators of OA. This study examined two tear biomarkers of inflammation: tear IgE and MMP-9, generating novel and confirmatory findings that contribute meaningfully to the evolving understanding of tear biomarkers in OA among hay fever sufferers.

The tear IgE and its correlation with subjective symptoms and QoL noted in this study are comparable to previous literature on the correlation with tear IgE to clinical signs, symptoms, and QoL [5,23,24,30,31,32,33,34,35,36,37,38,39,40,41,42]. However, the following important and novel contributions of this research were identified from the current study. It unraveled a previously unknown correlation of tear IgE with LWE apart from the other known correlation with palpebral roughness and bulbar redness [22,31]. Unlike previous literature, which did not find a correlation of tear IgE with participants who presented with a history of rhinitis [42], this study found a strong correlation between self-reporting of hay fever and tear IgE (rho = 0.68, *p* < 0.001) using i-ImmunDx^TM^ Analyzer, and a moderate correlation with tear IgE and lower LWE (rho = 0.41, *p* = 0.01). The predictive ability of tear IgE to differentiate hay fever sufferers from healthy controls, as mentioned in Table S3, indicates its usefulness in a routine clinical setup. Additionally, the eye rubbing frequency score, MiniRQLQ, QUICK, palpebral roughness, bulbar redness, and lower LWE are discriminators to differentiate between the hay fever group and healthy controls. This suggests that tear IgE and LWE can serve as an additional, and significant, diagnostic tool, complementing symptom scores, QoL scores, eye rubbing frequency score, palpebral roughness, and bulbar redness in the diagnosis of OA.

MMP-9 is a proteolytic enzyme that is involved in wound healing and inflammation, and is known to be involved in dry eye pathogenesis by disrupting the epithelial tight junctions and barrier functions [27,43,44]. As OA is also an inflammatory condition, we explored the potential of MMP-9 in predicting OA among hay fever sufferers. However, similar to the findings from previous literature [45], MMP-9 values in this study did not show a statistically significant difference between the hay fever and healthy control groups. However, we found an increase in the levels of MMP-9 among the hay fever group, when compared to HCs, a possible indicator of disrupted barrier function on the corneal epithelium due to friction. Changes in MMP-9 are often noticed in people with keratoconus [26,46], and it would be interesting for future researchers to investigate whether MMP-9 could be utilized as a precursor for the early detection of pre-keratoconic patients to halt the progression of corneal epithelium disruption. Our study population did not include anyone with keratoconus, and thus our hay fever group may not have yet progressed to such a severe epithelial tight junction disruption, as demonstrated by non-significant MMP-9 levels between the hay fever and healthy control groups. Hence, this study could not address the usefulness of MMP-9 as a tear biomarker in OA among hay fever sufferers. Focus has to be given to the exploration of other biomarkers, such as IL-33, IL-9 [47], Fibromodulin (FMOD) [18], IL-15 [48], IL-1, and growth factors [49], in future studies to develop a real-time tool for detecting the progressive disruption of tight junctions in allergic inflammations. This can be further extended to find the relationship between these biomarkers and clinical findings, especially corneal staining, which occurs due to disruption of the tight junctions in a much larger cohort.

### 3.2. Friction and Eye Rubbing

A particularly important finding from this study is the novel observation of significantly greater lower LWE among hay fever sufferers, when compared to HCs. This is the first study to demonstrate such an association in the context of OA.

This finding suggests a previously unrecognized mechanism in which increased friction between the lower eyelid margin and conjunctiva, likely due to repetitive eye rubbing and heightened ocular surface sensitivity, contributes to epithelial damage of lid margins among hay fever sufferers, while upper LWE has been documented in dry eye disease [43,50,51] and contact lens disorders [52,53]. Identification of lower LWE as a feature of OA opens an exciting new avenue for clinical diagnostic evaluation and understanding of the inflammatory cascade. This could also be largely because of the co-existence of dry eye [29,54] among OA patients.

LWE is believed to be caused by the mechanical trauma of the marginal conjunctiva of the upper eyelid on the ocular surface during blinking, resulting from inadequate lubrication at the lid wiper and constant rubbing [12]. LWE is an indicator of loss of homeostasis [29], and any morphological change can be a result of tear film disruption or signs of dry eye. Alternatively, the LWE of the lower lid seen in OA patients, as in the current study, might be an indication of the effect of eye rubbing and the impact of constant friction on the ocular surface. This is the first report on this association between lower LWE and OA. The relationship between tear IgE levels and the severity of LWE remains insufficiently studied. However, the mechanism could be mechanical stress due to increased IgE levels that trigger ocular itch. Future research is warranted to further clarify this association. These damaged epithelial cells may serve as an origin for the release of inflammatory mediators into the tear film, amplifying ocular discomfort and exacerbating allergic symptoms [8,18,42].

This finding not only enhances our understanding of OA pathophysiology but also highlights lower LWE as a practical and observable clinical sign. Its ease of detection and its significant positive correlation with tear IgE (rho = 0.41, *p* = 0.01), bulbar redness (rho = 0.60, *p* = 0.01), QUICK score (rho = 0.47, *p* = 0.03), MiniRQLQ score (rho = 0.51, *p* = 0.006), and upper LWE grade (rho = 0.55, *p* = 0.004), as mentioned in Table S2, suggest that LWE could be seamlessly integrated into routine clinical assessments, empowering clinicians with greater diagnostic confidence and expanding the scope of OA evaluation in everyday practice.

In the current study, eye rubbing was one of the main presenting symptoms (around 87.5%), as shown in Figure S2, along with nasal symptoms; however, two participants reported no eye rubbing. It is therefore important to educate hay fever sufferers on the long-term damaging effects of eye rubbing on the ocular surface.

The histogram in Figure S3 also shows that most of the hay fever sufferers rub their eyes for a few seconds, most probably the outside corner of the eyelids, in a soft fashion. This study did not question participants on rubbing of the specific areas of the upper or lower eyelid, which might have provided insight into any possible correlation between LWE of the lower eyelid and the area rubbed the most. Future studies are warranted to investigate any such correlations.

However, the potential contribution of co-existing dry eye conditions among hay fever participants cannot be ruled out and represents a limitation of the current study, particularly given the modest sample size. Co-existence of dry eye might have influenced the clinical signs and symptoms of participants. Future research should include dry eye diagnostics to better isolate hay fever-specific changes. The limited sample size made it difficult to perform further regression analysis to explain the true predictive ability of IgE among hay fever sufferers.

To summarize, this study proposes the practical diagnostic toolkit, called the OcuAllergy Diagnostic Toolkit (OADT), which integrates symptom questionnaires, clinical signs, and tear biomarkers, consisting of:

**Subjective findings**:

1. Self-reporting of hay fever
2. Eye rubbing frequency score
3. QUICK
4. MiniRQLQ

**Diagnostic marker:**

5. Tear IgE biomarker

**Clinical findings:**

6. Bulbar redness
7. Palpebral roughness
8. Lid wiper epitheliopathy

The 8-step OADT offers clinicians a comprehensive, evidence-based approach to improve OA recognition and diagnosis in hay fever sufferers, supporting earlier intervention and better patient outcomes. Future studies are recommended in a larger cohort to understand the usefulness of the OADT and evaluate its sensitivity and specificity in diagnosing OA among hay fever allergy sufferers. Studies involving the severity rating of OA (as mild, moderate, and severe OA) based on tear IgE is also recommended to understand the occurrence of signs and symptoms based on the severity of the disease.

## 4. Materials and Methods

Deakin University Human Research Ethics Committee (DUHREC) approved this study (Approval No. 2024/HE000250), and the study adhered to the principles outlined in the Declaration of Helsinki. This cross-sectional comparative study recruited 46 participants from Victoria, Australia, during the peak pollen season from November (spring) 2024 to January (summer) 2025. Eligible participants were aged between 18 and 50 years and were recruited via flyers.

The participants were classified into two groups: those who self-reported having hay fever and those who were healthy controls (HCs). Healthy controls were those who had never experienced symptoms of hay fever or any type of allergy. Exclusion criteria included the presence of any type of systemic illnesses, such as autoimmune disorders, lifestyle diseases, and Sjogren’s syndrome. History of eye surgery, contact lens use, and any use of medication that could affect the ocular surface were also excluded. Additionally, those who reported no history of hay fever but reported a history of perennial allergies due to pets or mold were also excluded from the analysis, as the focus of this research is OA in SAC, resulting from hay fever.

### 4.1. Symptom Assessment and Questionnaire-Based Clinical Measures

Participants first completed a screening questionnaire, which included demographic details and hay fever-specific questions, such as the presence of eye rubbing, style of eye rubbing, intensity of eye rubbing, and area rubbed, family history of allergies, and use of any medications for hay fever. In addition, validated questionnaires including the Quality of Life of Children with Allergic Keratoconjunctivitis (QUICK) questionnaire [30,55] and the Mini-Rhinoconjunctivitis Quality of Life Questionnaire (MiniRQLQ) [55,56] were administered to assess the impact of ocular and nasal symptoms on participants’ quality of life.

The QUICK questionnaire consists of 16 items, with the first 12 questions assessing the subjective severity of OA symptoms over the past two weeks, and the remaining 4 evaluating the social impact of allergic conjunctivitis. For this study, only the first 12 symptom-related questions were asked and analyzed, as this method has been used for this purpose in previous research [18]. Each of the 12 QUICK items has three possible response options: Never (1 point), Sometimes (2 points), and Always (3 points). The QUICK scores were calculated using the following formula:

(1) $$QUICK\;Score=\;\frac{\left[\Sigma subscale\;-\;12\right]}{\left[36-12\right]}\times 100$$

The QUICK scoring system using the formula above produces values from 0 to 100, where 0 indicates no symptoms and 100 indicates the most severe symptoms.

The MiniRQLQ was used to assess the quality of life among individuals with allergic symptoms. It consists of 14 items, each addressing how troubled participants have been over the last week due to nasal or ocular symptoms. Participants rated each item on a 7-point scale, ranging from Not Troubled (0 points) to Extremely Troubled (6 points), and average scores were calculated for analysis.

Additionally, eye rubbing frequency score was evaluated separately using a 5-point Likert scale, where 0 indicated No Eye Rubbing and 4 indicated Constant Rubbing [19].

### 4.2. Laboratory Biomarkers: Tear IgE and MMP-9

Tear samples were collected using a microcapillary fluid collector (Seinda Biomedical Corp, Guangzhou, China), following our established and routinely used protocol [23,31,42]. A total of 2.2 µL of unstimulated tears was collected from the right eye. All participants were instructed to tilt their head towards the side of the eye being measured, to look in the opposite direction at a target, and to blink normally throughout the test. The microcapillary tube was gently placed near the lateral canthus, and tears were collected by capillary action. The samples were loaded immediately onto the IgE or MMP-9 test cards, respectively, and followed with three drops of reaction buffer solution, provided in the respective kits. This allowed for real-time analysis without the risk of evaporation or the need for storage. The test cards were left flat and undisturbed for 15 min to allow reaction before being inserted into the i-ImmunDx^TM^ Analyzer (Seinda Biomedical Corp, Guangzhou, China). Quantitative values of tear IgE and MMP-9 levels were obtained for all participants. Tear samples were collected and processed by an experienced examiner under controlled conditions. Reflex tearing was minimized by giving the participants frequent breaks, reducing room illumination, and avoiding contact of the microcapillary tube with the eyelids or conjunctiva.

### 4.3. Clinical Measurements

A comprehensive slit lamp examination was conducted to assess ocular surface health. Palpebral roughness was evaluated using fluorescein dye and was graded according to the Brien Holden grading scale (0–4) [57]. The lid wiper staining was captured using the slit lamp camera after installation of lissamine green dye, and LWE was graded using the validated Photographic Lid Wiper Epitheliopathy (PLWE) grading scale [58]. The Oculus Keratograph K5 (Oculus, Inc., Wetzlar, Germany) automated measurements for bulbar redness grading and non-invasive tear break-up time were used for analysis. A single experienced optometry investigator performed all clinical assessments to ensure consistency with the technique.

In vivo confocal microscopy was performed using the Heidelberg Retinal Tomography (HRT)-Cornea module (Heidelberg Engineering, Franklin, MA, USA). Single-point images were acquired from the central cornea and temporal conjunctiva. The presence and absence of dendritic cells (DC) were identified by the investigator. The corneal and conjunctival DCs were defined as highly reflective cells with branching dendritic morphology, located at a depth of 50 to 70 μm at the level of the basal nerve plexus layer in the cornea [59], while conjunctival DCs were identified at a depth of 20–70 μm from the surface [60].

### 4.4. Statistical Analysis

Data were analyzed using SPSS (version 30) (Statistical Package for the Social Sciences, SPSS Inc., IBM, New York, NY, USA). The normality of the data was assessed using the Shapiro–Wilk (S-W) test. Tear IgE and MMP-9 values were log-transformed to base 10 before statistical testing to ensure normal distribution. Descriptive statistics were employed to calculate the median (IQR) for tear IgE, MMP-9, symptoms and signs, eye rubbing frequency score, and other variables. Correlation analyses were performed using the Spearman correlation to investigate the association between subjective scores and clinical signs. To adjust for multiple comparisons, the Benjamini–Hochberg false discovery rate (FDR) procedure was performed. An adjusted *p*-value (q-value) of less than 0.05 was considered statistically significant. Discrimination and predictive ability of test/test combinations were analyzed by the Receiver Operating Characteristic Curve (ROC), and the Area Under the Curve (AUC) is reported.

## 5. Conclusions

For the first time, this study has reported the importance of LWE and tear IgE levels as a potential diagnostic clinical sign while diagnosing OA. This study also highlights the usefulness of tear IgE in a clinical setup, as has been confirmed by reporting its significant correlation with symptom scores, eye rubbing frequency score, QoL, and lower LWE. This study is able to, for the first time, deliver a toolkit inclusive of subjective and objective findings for health care practitioners, including immunologists, while managing hay fever sufferers to easily diagnose OA. These can aid clinicians in providing early diagnosis and appropriate management of OA, without which it may progress to more severe eye conditions, such as keratoconus.

## Figures and Tables

**Table 1:** **Descriptive statistics and group comparisons:** Comparison of clinical symptoms and signs between the hay fever and healthy control groups.

Clinical Measure	Hay Fever	Healthy Controls	*p* Value
	(n = 16)	(n = 17)	
	Median (IQR)	Median (IQR)	
*Questionnaire scores*			
QUICK (0–100)	29.2 (17.6)	0.0 (12.5)	<0.001 *
MiniRQLQ (0–6)	3.1 (1.15)	1.30 (0.6)	<0.001 *
*Screening questions*			
Eye rubbing frequency score (0–4)	1 (1)	0 (1)	0.001 *
Eye rubbing (1-Yes, 0-No)	1 (0)	0 (1)	0.001 *
*Tear biomarkers*			
IgE IU/mL	2.03 (16.4)	0.15 (0.2)	<0.001 *
MMP-9 ng/mL	28.72 (63.9)	26.40 (174.8)	0.95
*Clinical signs*			
Bulbar Redness (0–4)	0.90 (0.4)	0.60 (0.2)	0.05
Palpebral Roughness (0–4)	2 (0.75)	1 (1.5)	<0.001 *
DC (1-Present/ 0-Absent)	1 (1)	0 (0.75)	0.038 *
NIKBUT (seconds)	9.24 (7.4)	9.11 (8.7)	0.99
LWE Upper Lid	2 (1.5)	2 (1)	0.15
LWE Lower Lid	2 (1.5)	1 (1)	<0.001 *

* Statistical significance: * *p* value < 0.05; Mann–Whitney U test; DC: Dendritic cells, LWE: Lid wiper epitheliopathy, NIKBUT: Non-invasive tear break-up time.

## Data Availability

All the data are provided in the article.

## Supplementary Materials

The following supporting information can be downloaded at: https://www.mdpi.com/article/10.3390/ijms26189116/s1.

## Abbreviations

The following abbreviations are used in this manuscript:

OA	Ocular Allergy
HCs	Healthy Controls
AC	Allergic Conjunctivitis
AR	Allergic Rhinitis
SAC	Seasonal Allergic Conjunctivitis
PAC	Perennial Allergic Conjunctivitis
VKC	Vernal Keratoconjunctivitis
AKC	Atopic Keratoconjunctivitis
GPC	Giant Papillary Conjunctivitis
CBC	Contact Blepharoconjunctivitis
QUICK	Quality of Life of Children with Allergic Keratoconjunctivitis
MiniRQLQ	Mini-Rhinoconjunctivitis Quality of Life Questionnaire
QoL	Quality of Life
LWE	Lid Wiper Epitheliopathy
OADT	OcuAllergy Diagnostic Toolkit
AAO	American Academy of Ophthalmology
ASCIA	Australasian Society of Clinical Immunology and Allergy
HRT	Heidelberg Retinal Tomography
POCT	Point of Care Test
DUHREC	The Deakin University Human Research Ethics Committee
AUC	Area Under the Curve
ROC	Receiver Operating Characteristic Curve
