# Supplementary material for: Exploring the Diagnostic Utility of Tear IgE and Lid Wiper Epitheliopathy in Ocular Allergy Among Individuals with Hay Fever
Source: Int J Mol Sci. 2025 Sep 18;26(18):9116. doi: 10.3390/ijms26189116 (PMC12470265; doi:10.3390/ijms26189116)
Supplement: Supplementary file 1 [file ijms-26-09116-s001.zip › ijms-3854622-supplementary.pdf]

__Supplementary Materials__

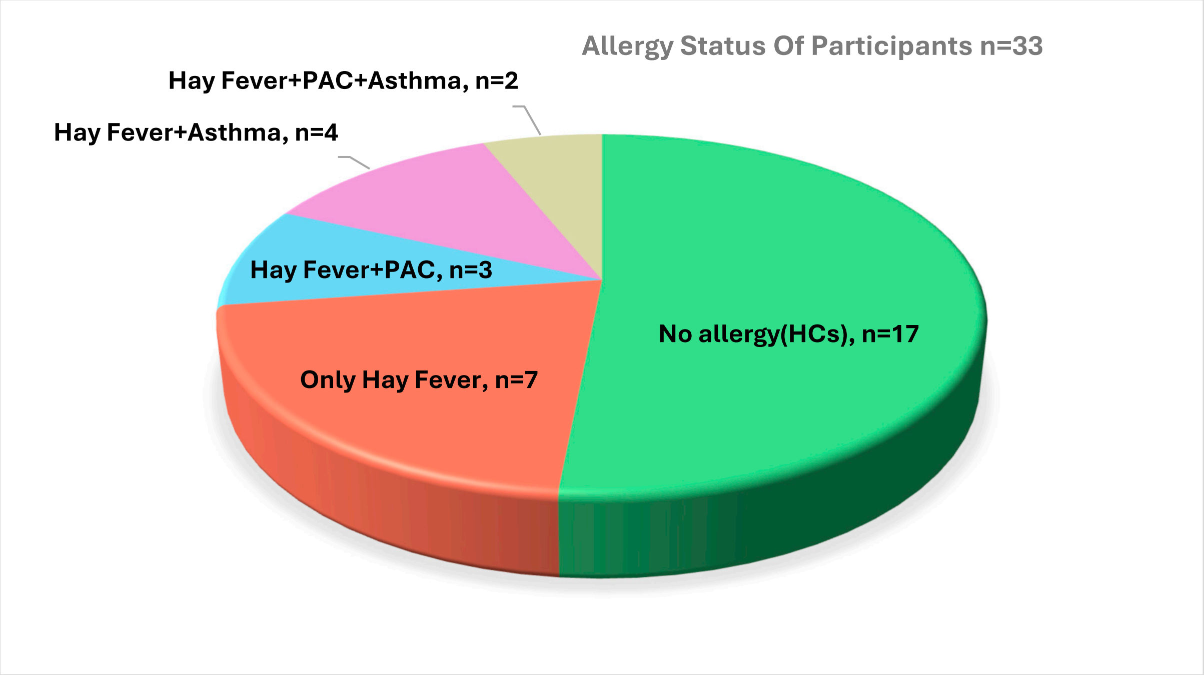

Figure S1: Distribution of participants by hay fever status. *The pie chart illustrates the final sample size (n=number of participants), consisting of 17 HCs and 16 hay fever sufferers. Some of the hay fever sufferers had comorbidities like PAC and Asthma, as indicated. PAC: Perennial Allergic Conjunctivitis; HCs: Healthy Controls.*

Table S1. ***Medications used by participants***. *The table shows the details of medication, including drug type use among the hay fever group to relieve symptoms (n=14), categorized by delivery method and pharmacological class. The "n" indicates how many participants used each medication; some reported using more than one medication.*

Category	Nasal Sprays	Eye Drops	Oral Medications	Inhalers
**Steroids (S)**	Mometasone furoate, n = 1<br><br>Budesonide, n = 1	Fluorometholone, n = 1		Fluticasone propionate, n = 1
**Antihistamines (A)**			Loratadine n = 5<br>Fexofenadine hydrochloride, n = 8<br>Cetirizine, n = 7	
**Mast Cell Stabilizers (MCS)**				
**Combination**	Azelastine; Luticasone propionate, n = 5 (A + S)	Ketotifen, n = 1 (A + MCS)		
**Lubricants**		Systane n =1		
**Bronchodilators**				Salbutamol n = 3
**Other**			Herbal treatments n = 1	

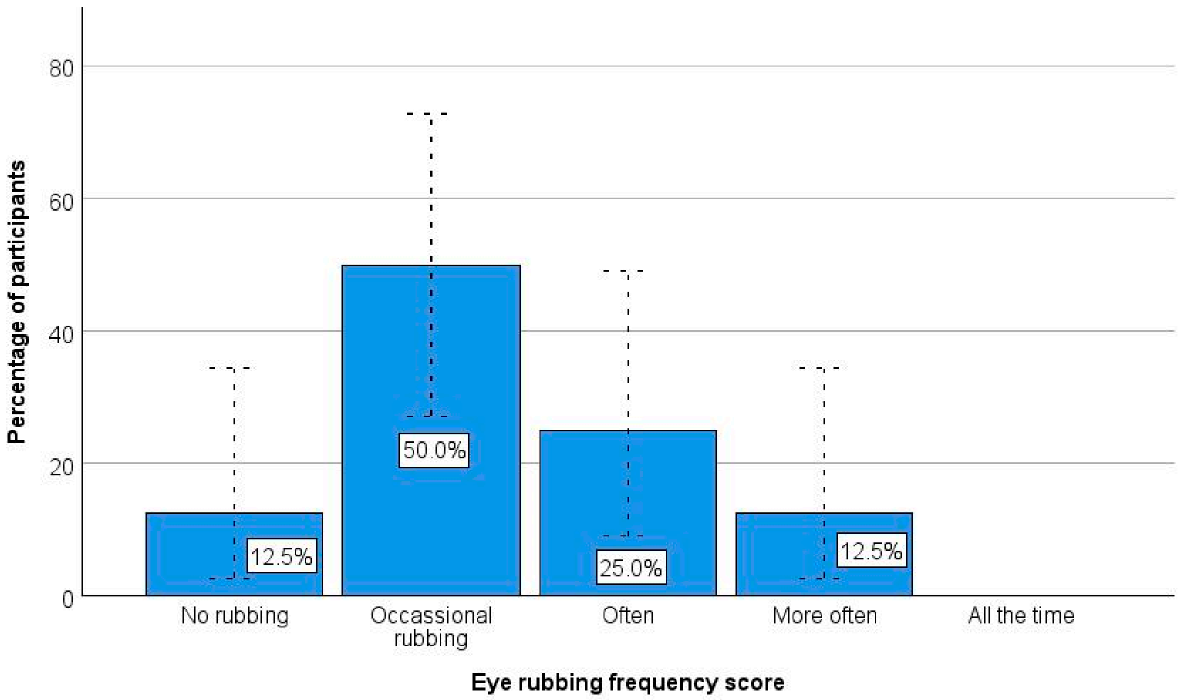

Figure S2: Eye rubbing frequency score for hay fever participants. *Frequency of eye rubbing distribution in patients with hay fever on a scale of 0 (No rubbing) to 4 (Rubbing all the time), n=16.*

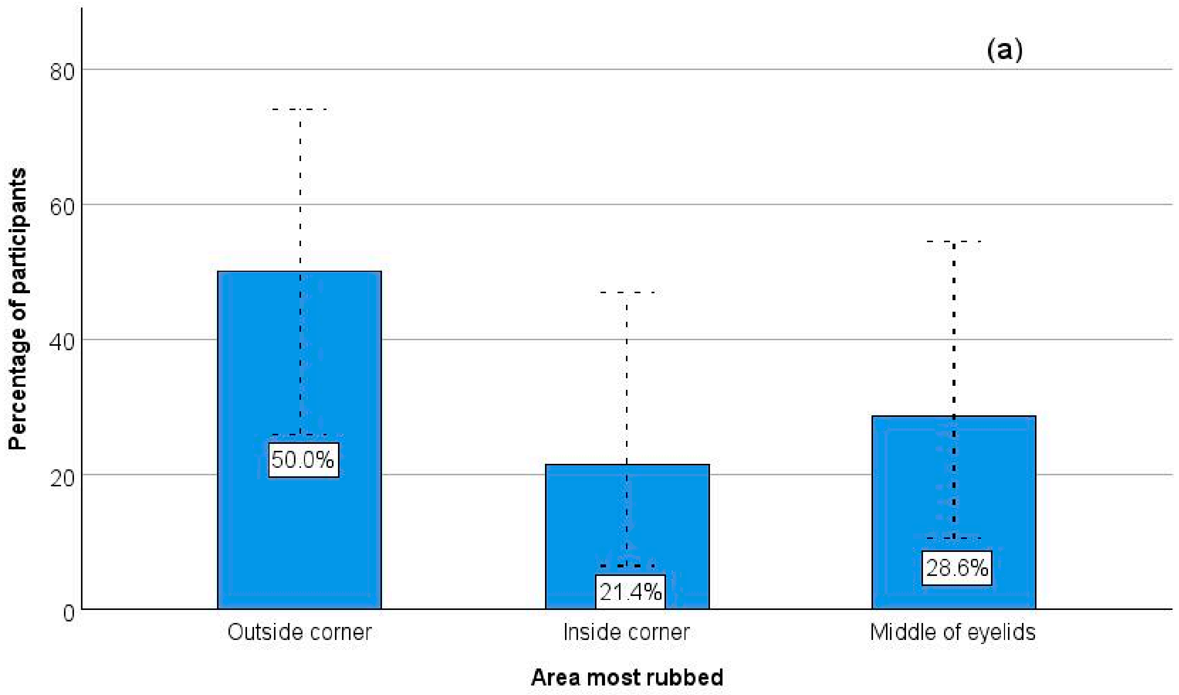

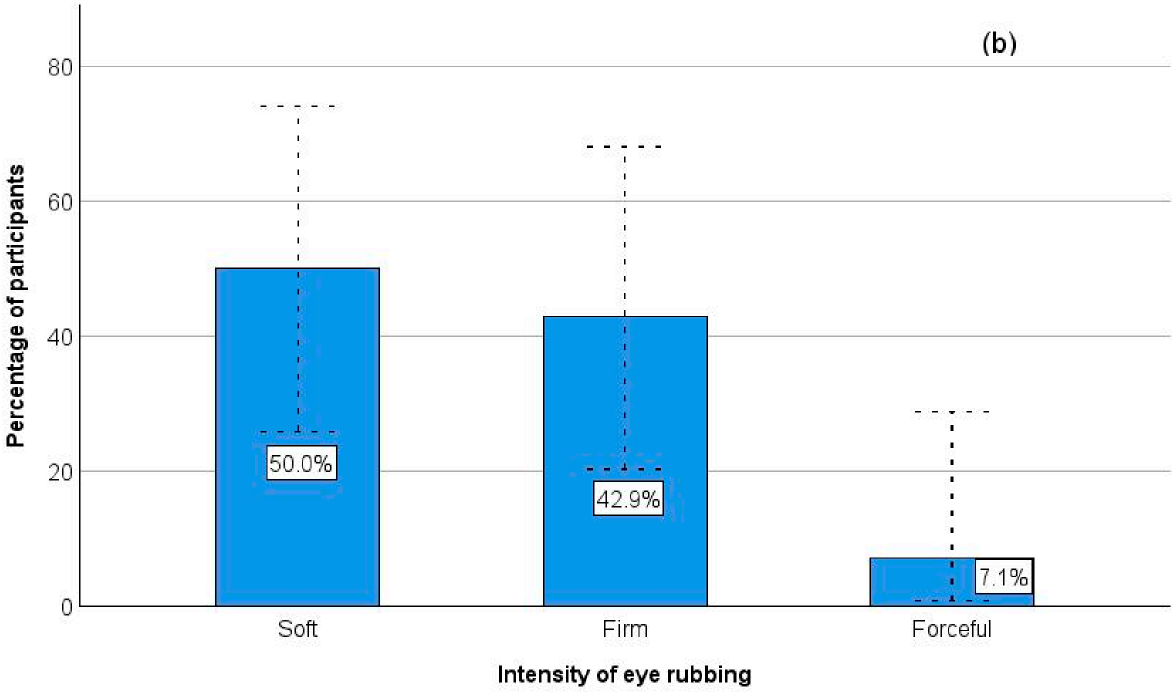

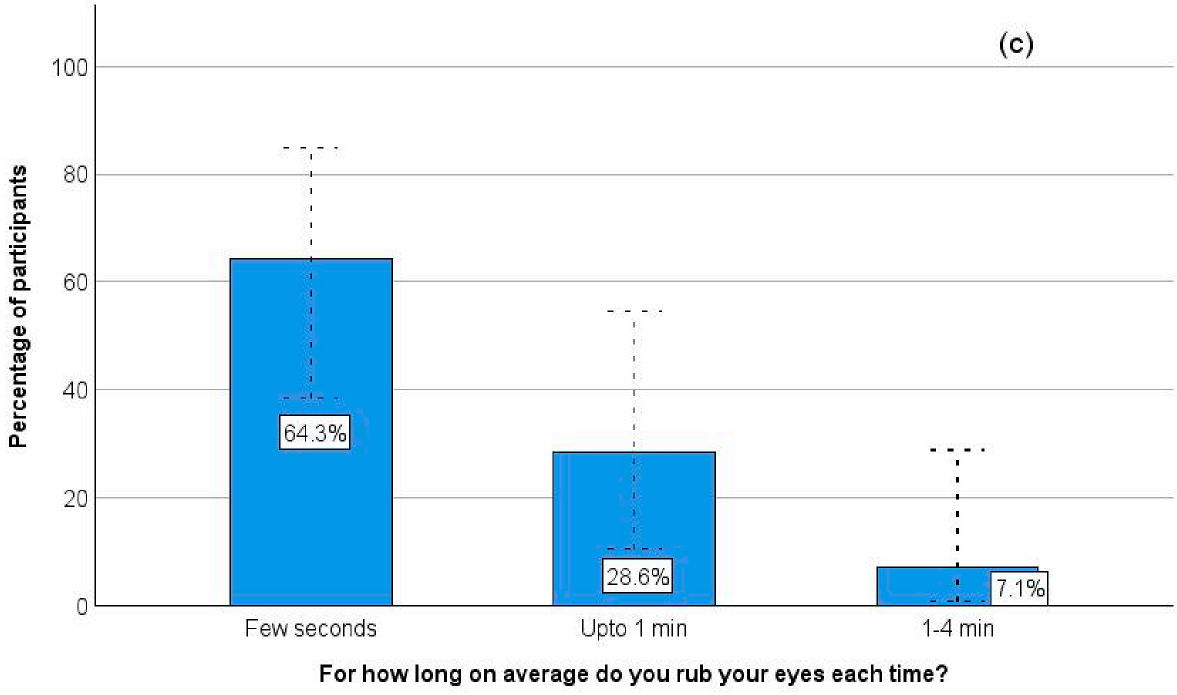

Figure S3 Characteristics of eye rubbing among hay fever sufferers. *The bar graph represents the area rubbed (a), intensity of eye rubbing (b) and duration of eye rubbing (c) among hay fever sufferers n=16*

Table S2: Correlation analysis. *Spearman correlation between self-reporting of hay fever, tear IgE, MMP-9, clinical measurements of conjunctival papillae, bulbar redness, tear break-up time, and lid wiper epitheliopathy, mini-RQLQ, eye rubbing frequency score, and QUICK scores. Those parameters with good correlation and are statistically significant are shown in bold. n=33 if otherwise specified due to missing data. Correlation coefficients in bold indicate significance levels were p< 0.05. P-values were adjusted for multiple comparisons using the Benjamini–Hochberg false discovery rate (FDR) procedure. An adjusted p-value (q-value) of less than 0.05 was considered statistically significant. Abbreviations: LWE: Lid Wiper Epitheliopathy, NIKBUT: Non-invasive keratometry break up time, DC: Dendritic Cells, UL Upper Lid, LL: Lower Lid*

	Self-reporting of hay fever	IgE levels	Eye Rubbing Frequency Score	MiniRQLQ	QUICK	Bulbar redness	Palpebral roughness	LWE_UL	LWE_LL	Tear MMP-9	NIKBUT	Presence of DCs
IgE levels	**0.68**											
Eye Rubbing Frequency Score	**0.60**	**0.63**										
MiniRQLQ	**0.78**	**0.6**	**0.54**									
QUICK	**0.74**	**0.55**	**0.60**	**0.84**								
Bulbar redness	0.36	0.26	0.13	0.21	0.30							
Palpebral roughness	**0.60**	0.28	0.30	**0.47**	**0.54**	**0.46**						
LWE_UL	0.28	0.30	0.20	0.15	0.09	0.41	0.24					
LWE_LL	**0.65**	**0.41**	0.22	**0.51**	**0.47**	**0.60**	0.40	**0.50**				
Tear MMP-9	0.01	0.02	-0.03	-0.10	0.12	0.37	0.15	0.07	0.20			

NIKBUT	0.003	-0.34	-0.20	0.007	0.02	-0.24	0.15	-0.03	-0.02	-0.40		
Presence of DCs	0.41	0.28	0.30	0.21	0.37	0.43	0.46	0.37	**0.54**	0.26	0.08	

Table S3: ***Area Under the Curve in OA vs HCs:*** *The table represents the area under the curve, while participants were grouped based on self-reporting of symptoms. Area under the ROC (AUC), P-value, 95% confidence interval of AUC, threshold (TH), sensitivity, and specificity. p value mentioned with an * indicates significance levels were <0.05*

**Test Result Variable(s)**	**AUC**	**p value**	**Asymptotic 95% Confidence Interval**		**TH**	**Sensitivity**	**1-specificity**
Tear_IgE	0.89	0.00*	0.71	1.08	0.03	0.90	0.85
Tear_MMP9	0.52	0.86	0.28	0.76	2.1	1.00	0.85
MiniRQLQ	0.96	0.00*	0.9	1.03	1.05	1.00	0.64
QUICK	0.98	0.00*	0.93	1.03	2.1	1.00	0.42
Eye rubbing frequency score	0.88	0.00*	0.73	1.03	0.5	0.90	0.28
Palpebral Roughness	0.85	0.00*	0.69	1	0.5	1.00	0.57
Bulbar Redness	0.74	0.03*	0.52	0.95	0.35	1.00	0.85
LWE_UL	0.64	0.24	0.41	0.86	0.5	1.00	0.92
LWE_LL	0.87	0.00*	0.72	1.02	0.5	1.00	0.57
Presence of prominent Dendritic Cells	0.66	0.17	0.43	0.88	0.5	0.60	0.28
Average NIKBUT	0.44	0.63	0.21	0.68	5.6	0.90	0.85

Table S4 ***Area Under the Curve based on tear IgE cut off 1 IU/mL:*** *The table represents the area under the curve, while participants were grouped based on tear IgE cut off value of 1 IU/mL. Area under the ROC (AUC), P-value , 95% confidence interval of AUC, threshold (TH), sensitivity, and specificity. p value mentioned with an * indicates significance levels were <0.05*

**Test Result Variable(s)**	**AUC**	**p value**	**Asymptotic 95% Confidence Interval**		**TH**	**Sensitivity**	**1-specificity**
Tear_MMP9	0.45	0.70	0.22	0.69	5.4	0.87	0.81
MiniRQLQ	0.90	0.00*	0.77	1.03	1.0	1.00	0.68
QUICK	0.95	0.00*	0.87	1.03	2.1	1.00	0.50
Eye rubbing frequency score	0.94	0.00*	0.85	1.03	0.5	1.00	0.31
Palpebral Roughness	0.76	0.01*	0.56	0.96	0.5	1.00	0.62
Bulbar Redness	0.62	0.33	0.38	0.87	0.35	1.00	0.87
LWE_UL	0.61	0.40	0.36	0.86	0.5	1.00	0.93
LWE_LL	0.78	0.002*	0.60	0.97	0.5	1.00	0.62
Presence of prominent Dendritic Cells	0.66	0.20	0.42	0.89	0.5	0.62	0.31
Average NIKBUT	0.367	0.260	0.136	0.598	4.6	0.870	1.000
